# Self-Assembly of Lipid Molecules under Shear Flows: A Dissipative Particle Dynamics Simulation Study

**DOI:** 10.3390/biom13091359

**Published:** 2023-09-07

**Authors:** Huan Zhang, Fan Pan, Shiben Li

**Affiliations:** 1 Department of Physics, Wenzhou University, Wenzhou 325035, China; 2 School of Data Science and Artificial Intelligence, Wenzhou University of Technology, Wenzhou 325035, China

**Keywords:** lipid molecules, phase diagrams, shear flow, self-assembly, dynamics process

## Abstract

The self-assembly of lipid molecules in aqueous solution under shear flows was investigated using the dissipative particle dynamics simulation method. Three cases were considered: zero shear flow, weak shear flow and strong shear flow. Various self-assembled structures, such as double layers, perforated double layers, hierarchical discs, micelles, and vesicles, were observed. The self-assembly behavior was investigated in equilibrium by constructing phase diagrams based on chain lengths. Results showed the remarkable influence of chain length, shear flow and solution concentration on the self-assembly process. Furthermore, the self-assembly behavior of lipid molecules was analyzed using the system energy, particle number and shape factor during the dynamic processes, where the self-assembly pathways were observed and analyzed for the typical structures. The results enhance our understanding of biomacromolecule self-assembly in a solution and hold the potential for applications in biomedicine.

## 1. Introduction

Lipid molecules usually possess an amphiphilic structure, characterized by a hydrophilic head chain and one or more hydrophobic tail chains. This unique property enables lipid molecules to undergo self-assembly, forming a diverse range of nanostructures in a solution [1,2]. These self-assembled structures have been widely used in biomedical applications. For example, lyotropic liquid crystal phases, self-assembled from monoglyceride molecules in solutions, have been utilized as drug carriers due to their ability to incorporate large quantities of drugs with varying properties [3]. Thus, the self-assembly of lipid molecules in solutions, whether with one or two tail chains, has received increasing attention in recent years. This interest stems from both the desire to comprehend the underlying self-assembly mechanisms and the potential applications that can be derived from them.

Phospholipid molecules, possessing two tail chains, are crucial constituents of phospholipid bilayers. In a solution, these molecules can self-assemble into bilayer membranes and other nanostructures, such as cylinders and vesicles. Numerous computer simulations and experiments have contributed to the understanding of the self-assembled microstructures of phospholipid molecules in solutions in the absence of shear flow [4,5,6,7,8,9,10,11,12,13,14,15]. There are many experiments contributed to the self-assembly of phospholipid molecules, including atomic force microscopy (AFM) and small angle X-ray scattering (SAXS) experiments. For example, AFM experiments have observed phase transitions in POPE and POPG membranes induced by temperature and ion concentration [4]. SAXS measurements have identified phase transitions between lamellar and inverted-hexagonal phases [7]. Dissipative particle dynamics (DPD) simulations utilizing coarse-grained (CG) models have investigated the effects of chain length on the self-assembly of phospholipid molecules in a solution, shedding light on the transition between different phases [14]. Moreover, molecular dynamics simulations at the all-atom level have examined bilayer membranes constructed from DPPC, DOPC and cholesterol, revealing the coexistence of liquid and gel or ripple phases in a binary mixture [15]. Consequently, investigations on biological membranes and other nanostructures under shear flow have attracted much attention [16,17,18,19,20,21,22,23,24,25,26,27]. For example, CG molecular dynamics simulations have shown that external shear flow applied to a lipid bilayer generates hydrodynamic forces, causing the phospholipid molecules to move in the direction of flow. When the shear rate exceeds a critical value, the membrane undergoes deformation perpendicular to the shear flow [19].

Lipid molecules possessing one head chain and one tail chain are also able to self-assemble into traditional phases in solutions, such as lamellar and cylindric phases, and a series of bicontinuous cubic phases with various symmetries [28,29,30,31,32,33,34,35,36,37]. For example, SAXS experiments have observed bicontinuous cubic phases with various symmetries (Ia3d, Pn3m and Im3m) of lipids with one head and one tail chain in aqueous solutions, such as monolaurin (ME), monoacidic (MV), monoolein (MO) and monokinetid (ML) [30,31]. The results showed that these Ia3d, Pn3m and Im3m bicontinuous cubic phases depend on the water concentrations and the system temperature. Moreover, lamellar crystalline phases have been observed for ML and ME molecules in aqueous solutions where the phase transitions occur due to the chain splay and the pressure and temperature of systems [29,30], but they differ from those found in phospholipid bilayers [38,39,40,41,42,43]. The nanomechanical properties of a cubic phase with Pn3m symmetry have been characterized using SAXS and AFM, revealing its response to topology and structure [36]. Recent experiments employing SAXS and broadband dielectric spectroscopy have reported phase transitions between the double gyroid (Ia3d) and double diamond (Pn3m), and between the latter and the reverse hexagonal phases, demonstrating temperature-dependent transitions [37]. DPD simulations have reported that lipid molecules can form various nanostructures, such as bilayer membranes and cylinders, in solutions depending on the lengths of tail chains. Phase diagrams have been constructed, and mixing assembly has been observed in the absence of shear flow [44]. However, recent DPD simulations demonstrated that shear flow can strongly affect the self-assembly of phospholipid molecules in a solution where the chain lengths are changed [14]. Similar to phospholipid molecules [27,45], shear flow affects the self-assembly of lipid molecules in a solution. Recent experiments on MO lipids have confirmed this effect, investigating the relationship between shear flow and structures [46].

The existing simulations and experiments inspired our investigation into the self-assembly mechanism of lipid molecules with one head and one tail chain under shear flow conditions and various lipid concentrations. In this study, we used the DPD method to investigate the self-assembly of lipid molecules with one head and one tail chain under shear flow. We consider two types of lipids and investigate the phase behavior induced by their tail chain lengths. Our focus is on the effect of shear flow and lipid concentration on the self-assembled structures, the phase diagram for the lipid molecules, and the structural formations observed during the dynamic processes. Section 2 introduces the method and model descriptions. Section 3 presents the results and discussion. Section 4 provides a summary of our findings.

## 2. Models and Methods

### 2.1. Simulation Method

The DPD method is a powerful simulation technique that captures the dynamic behavior of complex fluids and soft materials by representing a group of atoms as particles [15,47,48,49,50]. This method, initially proposed by Hoogerbrugge and Koelman in 1992 [51] and later improved by Basan [52], has proven effective in studying various systems. In our simulations, we employed the DPD method, and we will provide a brief overview of the main equations used. Consider a system of *N* particles with constant volume *V* and temperature *T*. In DPD, the forces acting between the *i*-th and *j*-th particle types can be categorized into three types: conservative force $F_{ij}^{C}$, dissipative force $F_{ij}^{D}$ and random force $F_{ij}^{R}$. The total force acting on the *i*-th particle, $r_{i}$, can be expressed as

(1) $$F_{i}=\sum_{i=1,i\neq j}^{N}\left(F_{ij}^{C}+F_{ij}^{D}+F_{ij}^{R}\right).$$

The movement of the *i*-th particle follows Newton’s laws, where $dr_{i}/dt=v_{i}$ and $m_{i}\;dv_{i}/dt=F_{i}$. Here, $r_{i}$, $v_{i}$ and $m_{i}$ denote the position, velocity and mass of the *i*-th particle, respectively. $F_{ij}^{C}$, $F_{ij}^{D}$ and $F_{ij}^{R}$ can be expressed as [53,54,55]

(2) $$F_{ij}^{C}=a_{ij}w\left(r_{ij}\right){\hat{r}}_{ij},$$

(3) $$F_{ij}^{D}=-\gamma w^{2}\left(r_{ij}\right)\left(v_{ij}\cdot {\hat{r}}_{ij}\right){\hat{r}}_{ij},$$

(4) $$F_{ij}^{R}=\sigma w\left(r_{ij}\right)\xi_{ij}{\left(\Delta t\right)}^{-1/2}{\hat{r}}_{ij},$$

where $a_{ij}$ is a parameter that represents the strength of the soft repulsive interaction between the *i*-th and *j*-th particles, $r_{ij}$ denotes the distance between the particles and ${\hat{r}}_{ij}$ is the unit vector pointing from particle *i* to particle *j*, $r_{ij}=$$\left|r_{i}-r_{j}\right|,{\hat{r}}_{ij}=\left(r_{i}-r_{j}\right)/r_{ij}$. The relative velocity between the particles is denoted by $v_{ij}$, $v_{ij}=v_{i}-v_{j}$. $\zeta_{ij}$ is a random number with mean 0 and variance 1 from a uniform random distribution with unit variance and Gaussian distribution. γ and σ denote the dissipation and noise intensity coefficients, respectively, which satisfy the relation $\sigma^{2}=2\gamma k_{B}T$. We note that the γ and σ are independent of the *i*-th and *j*-th particles, which differ from the parameters $a_{ij}$ and $\zeta_{ij}$. The weight function $w\left(r_{ij}\right)$ is defined as follows:

(5) $$w\left(r_{ij}\right)=\left\{\begin{array}{cc} 1-\frac{r_{ij}}{r_{c}} & r_{ij}<r_{c}\\ 0 & r_{ij}>r_{c} \end{array}\right.,$$

where $r_{c}$ is the cut-off radius in all simulations.

### 2.2. Lipid Molecule Model

**Figure 1:**
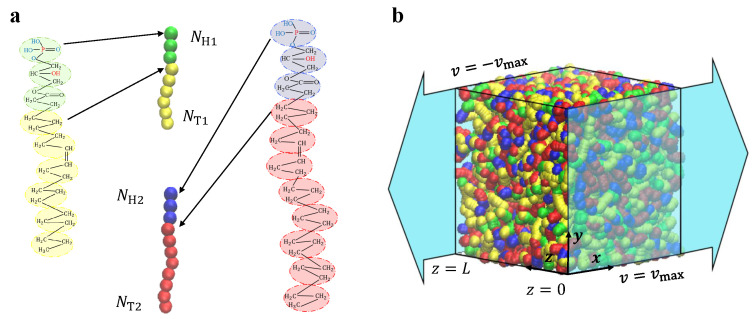
The lipid and shear flow models. (**a**) Coarse-grained models for the lipids with one head and one tail chain. The head particles of the type-I and type-II lipids are represented by green and blue beads, and the tail particles are represented by yellow and red beads, respectively. The chemical formulas of lipid molecules are displayed on both sides. (**b**) The shear flow is imposed on the system in the *x* direction, where the simulation box volume is L×L×L. The maximum velocities are applied at z=0 and z=L, and the velocity gradient is along the *z* direction.

DPD is a simulation technique based on particle dynamics that describes the dynamical processes. In our current simulations, we used a CG model for the lipid molecules, as shown in Figure 1a, where both the CG particles and chemical structures are listed. This lipid model is similar to previous DPD simulations, and we will provide a brief overview of the main properties involved [44]. The CG model represents many carbon atoms as particles that form linear chains to construct the head and tail linear regions [50], resembling the structure of lysophosphatidic acid [44]. Although phospholipid molecules are typically represented as a chain with a head and two chains in CG simulations [50,56], our model focuses on lipid molecules with one head chain and one tail. The head particles of the two lipid molecule types (type-I and type-II) are represented by green and blue beads, respectively, and the tail particles are represented by yellow and red beads. The experiment suggested that the chain length of the monoolein (MO) lipid molecule can be exactly changed by varying the number of carbon bonds [57]. This makes it realistic to change the chain length of lipid molecules in our DPD modeling. Meanwhile, when polymer chains are synthesized in experiments, these chains are dispersible, which can be described by the polydispersity index. These facts suggested that the current type-I and type-II lipids with different chain lengths are suitable.

To maintain the connectivity between particles throughout the simulations, we employed elastic harmonic forces [58,59]. The expression for the elastic harmonic force is

(6) $$F_{ij}=k_{s}\left(1-\frac{r_{ij}}{r_{s}}\right){\hat{r}}_{ij},$$

where $k_{s}$ is the spring coefficient, and $r_{s}$ is the equilibrium bond length. In our simulations, we used the parameters $k_{s}$ = 120.0 and $r_{s}=0.7$ $r_{c}$, which are consistent with previous work [60]. The antibonding ability of lipid molecules is achieved through an additional force known as the bending force, which arises from the harmonic binding of two consecutive bonds. The expression for the bending force is

(7) $$F^{\theta }=-\nabla \left[k_{\theta }{\left(\theta -\theta_{0}\right)}^{2}\right],$$

where $k_{\theta }$, $\theta_{0}$, and θ are the bending constant, equilibrium angle, and tilt angle, respectively. In our simulations, we set $k_{\theta }=6.0$ and $\theta_{0}=\pi$, which is consistent with previous DPD simulations [44,61]. Here, we note that the elastic harmonic force and bending force exist in the lipid chains to maintain the connectivity and rigidity of chains, which are different from the DPD forces in Equation (1).

### 2.3. Shear Flow Model

The reverse nonequilibrium method (RNEMD) is a widely used approach for calculating shear viscosity by connecting shear fields and transverse linear momentum fluxes [14,61,62,63,64,65,66,67,68,69]. In our simulations, we employed RNEMD to generate shear flow, as shown in Figure 1b. In this method, the shear rate (commonly known as shear strength) and momentum flux are expressed by the equations $\dot{\gamma }=\partial v_{x}/\partial z$ and $j_{z}\left(P_{x}\right)=-\eta \partial v_{x}/\partial z$, where η is a constant factor relating the momentum flux to the shear velocity [67]. The velocity has a component in the *x*, *y*, and *z* directions. When we calculate the partial differential of the *x* component of the velocity concerning *z*, we assume that the *y* and *z* components of the velocity are constants. The partial differential of momentum flux is treated similarly. In RNEMD, the momentum flux is applied to the system in an unphysical manner [67]. Specifically, the simulation box is divided into multiple flat plates along the flow field direction, with two symmetric planes experiencing shear flow in the *x*-direction and velocity gradients in the *z*-direction. The particles in the system can be divided into an infinite number of flat plates along the *z*-direction, where particles with z=0 are driven in the *x*-positive direction, and particles with z=L are driven along the *x*-negative direction [70]. The potential energy and energy of the system are conserved when the particles have the same mass and no change in position by finding particles with the maximum and minimum momentum along the *x*-direction, allowing for momentum exchange [71]. The shear velocity distribution shows a distinct linear relationship along the *z*-axis, verifying the feasibility of this method [14,61]. As the momentum exchange exhibits periodicity, the sum of $\Delta P_{x}$ during the simulation corresponds to the total momentum of the system. In the non-equilibrium state, the rate of momentum exchange through the unphysical method matches the rate of momentum return to the fluid through friction. Thus, the momentum flux can be expressed as

(8) $$j_{z}\left(P_{x}\right)=\frac{P_{x}}{2tL_{x}L_{y}},$$

where *t* is the exchange time, and $L_{x}$ and $L_{y}$ represent the lengths of the two sides of the periodic box in the *x* and *y* directions, respectively, [67]. The exchange time *t* is adjusted by controlling the magnitude of the momentum flux to achieve different shear flow strengths, where t=MΔt with *M* is a multiple of the time step Δt. For the shear rate, we considered two different shear flow cases: weak and strong shear flows, corresponding to M=6 and M=1, respectively. The corresponding shear rates are $\dot{\gamma }=$ 0.073 $\tau^{-1}$ and $\dot{\gamma }=0.168$ $\tau^{-1}$, which are consistent with previous simulations [61]. We note that this definition is relative, and in general, the strong shear flow can easily deform the nanostructures.

### 2.4. Simulation Parameters

The simulation parameters used in our study are set as follows (Table 1). We chose to simulate the selfassembly of lipid molecules in water using a particle number density of ρ=3 and a DPD particle volume of $V_{P}=0.03\;{\mathrm{nm}}^{3}$ based on previous studies [14,50,72,73]. The cut-off radius $r_{c}$ is determined using the equation $r_{c}={\left(\rho V_{P}\right)}^{1/3}$, resulting in an approximate cut-off radius size of 0.5nm for the lipid–water mixture in our study [44,61]. For simplicity, we used dimensionless parameters for the DPD calculations. In this model, we assume that all particles have the same mass, denoted as $m_{i}\;=$ *m*, and define *m* as the unit mass. We then use $r_{c}$ as the unit length, $k_{B}T$ as the unit energy and τ as the unit time [44]. Selecting an appropriate time step in the simulation is crucial, which is related to the velocity Verlet algorithm. In our simulations, we chose a time step of Δt=0.01 τ, similar to previous simulations [55]. The time τ can be calculated as [74]

(9) $$\tau =r_{c}\sqrt{m/k_{B}T}.$$

Based on in-plane diffusion constants from experiments, we obtained τ=1.88 ns, yielding Δt=0.0188 ns [75,76].

In DPD simulations, it is important to determine the interaction parameters between particles. These DPD interaction parameters are related to Flory–Huggins interaction parameters, which can be expressed as $\chi =0.286\left(a_{ij}-a_{ii}\right)$ [77]. This mutual repulsion parameter $a_{ij}$ corresponds to the thermodynamic incompatibility of the actual substance, which can be derived from experimentally measurable properties such as compression coefficients, solubility parameters, etc., thus constructing the bridge between experimental observations and DPD simulations. In the current DPD simulations, the repulsion parameter between identical particles is set to 25, and the repulsion parameter between different particles is set to either 100 or 40. For the head and tail chains of the lipid molecules, we chose parameters consistent with experimental results, as listed in Table 2 [31,32,33,78]. Specifically, the repulsion parameter between the head particle and water particle is set to 40, and the repulsion parameter between the tail particle and water particle is set to 100. This choice yields Flory coefficients of approximately 4.29 for the head particle–water interaction and 21.42 for the tail particle–water interaction [44]. Consequently, the head particle weakly interacts with water, whereas the tail particle exhibits a strong separation from water.

**Figure 2:**
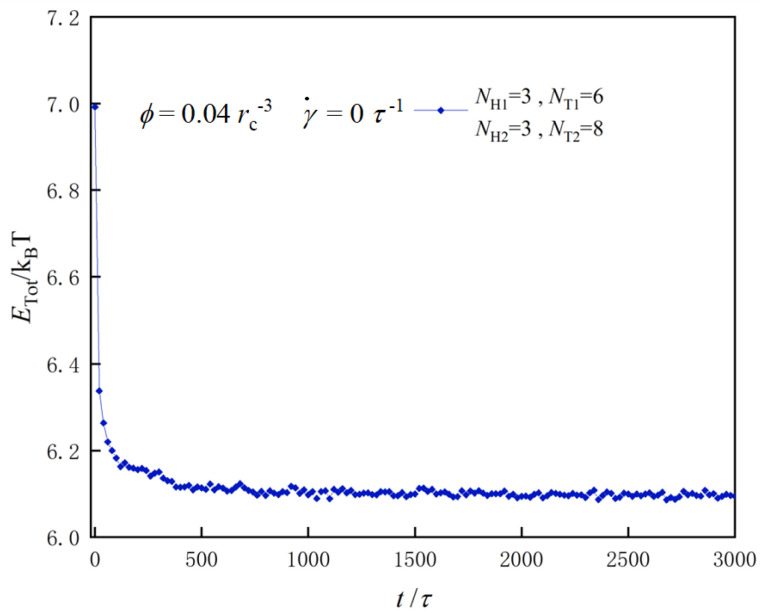
An example for obtaining the stable state with parameters of $N_{H1}=3,N_{H2}=3,N_{T1}=6$, and $N_{T2}=8$, in which the total energy varies as a function of time steps.

DPD simulations were performed in an NVT ensemble using the massively parallel atomic/molecular simulator (LAMMPS) [79]. The system reached a stable state after running for 3000 τ time steps, as shown in Figure 2. We compared the system energy across several different states and selected one with the lowest energy to determine the most appropriate initial input state for the simulations [44,61]. To optimize the box size effect in the DPD simulation, we varied the size of the simulation boxes from 25 $r_{c}$ to 35 $r_{c}$. Finally, we chose a simulation box with a size of 30 $r_{c}$, resulting in a cubic volume of V=30 $r_{c}\;\times$ 30 $r_{c}\;\times$ 30 $r_{c}$ for the current simulations.

## 3. Results and Discussion

**Figure 3:**
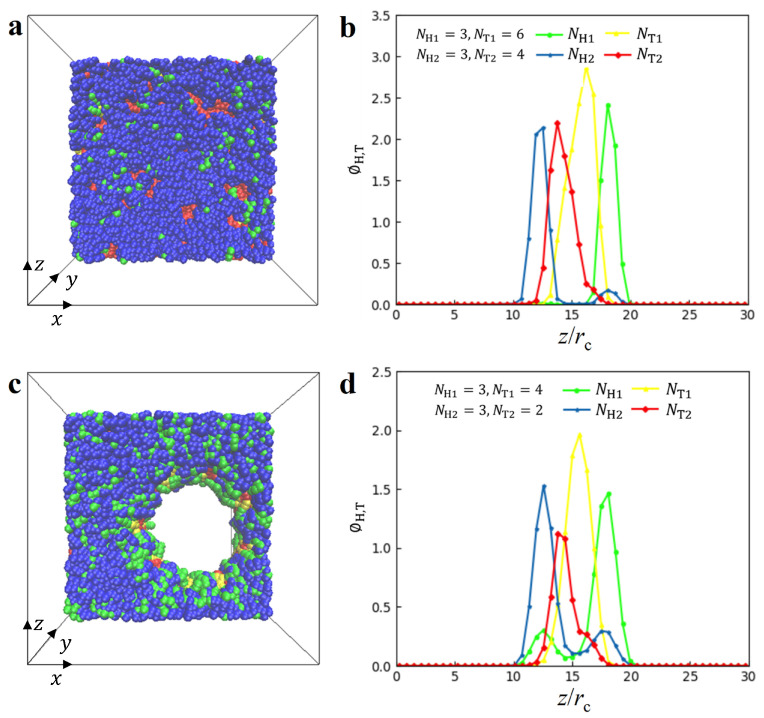
Representative DL and PDL nanostructures. (**a**) The top view and (**b**) density distribution along z direction for DL structure with parameters of $N_{H1}=3,N_{H2}=3,N_{T1}=6$ and $N_{T2}=4$ when ϕ = 0.08 $r_{c}^{-3}$, $\dot{\gamma }=0$ $\tau^{-1}$. (**c**) The top view and (**d**) density distribution along *z* direction for PDL structure with parameters of $N_{H1}=$$3,N_{H2}=3,N_{T1}=4$ and $N_{T2}=2$ when ϕ = 0.08 $r_{c}^{-3}$, $\dot{\gamma }=0.073$ $\tau^{-1}$.

**Figure 4:**
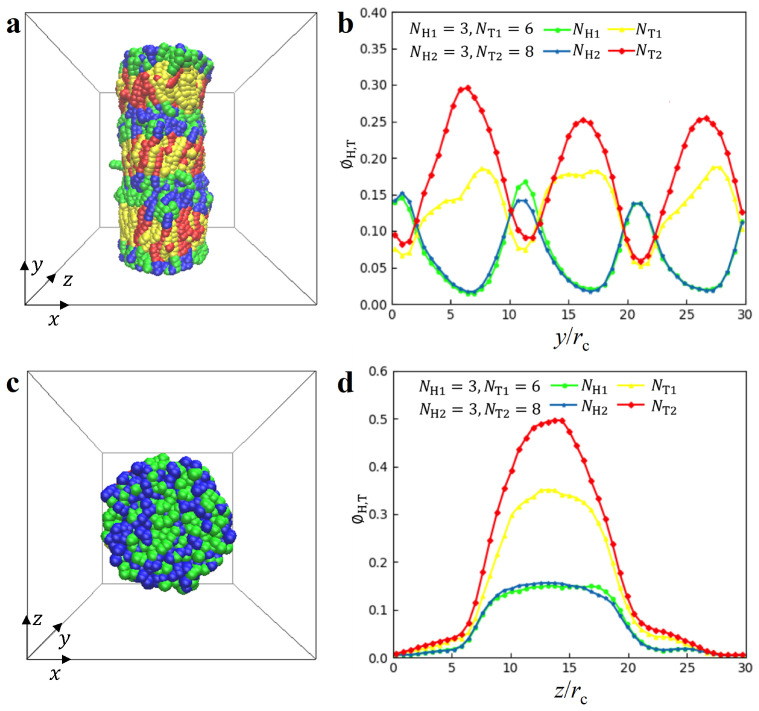
Representative HD nanostructures with parameters of $N_{H1}=3,N_{H2}=3,N_{T1}=6$ and $N_{T2}=8$ when ϕ = 0.04 $r_{c}^{-3}$, $\dot{\gamma }=0$ $\tau^{-1}$. (**a**) The side view and (**b**) density distribution along *y* direction. (**c**) The top view and (**d**) density distribution (**d**) along *z* direction.

**Figure 5:**
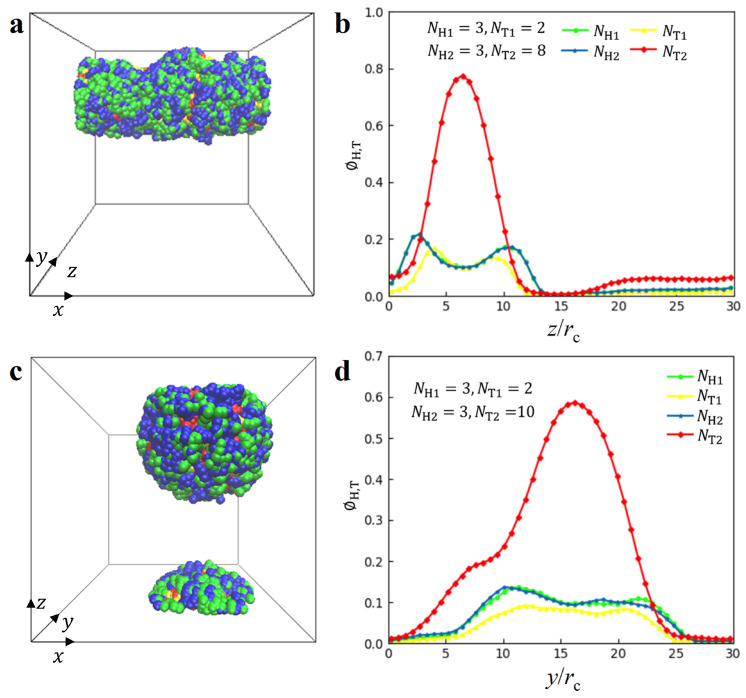
Representative micelle nanostructure with parameters of $N_{H1}=3,N_{H2}=3,N_{T1}=2$ and $N_{T2}=8$ when ϕ = 0.08 $r_{c}^{-3}$, $\dot{\gamma }=0$ $\tau^{-1}$ and vesicle nanostructure with parameters of $N_{H1}=3$, $N_{H2}=3,N_{T1}=2$ and $N_{T2}=$ 10 when ϕ = 0.08 $r_{c}^{-3}$, $\dot{\gamma }=0.073$ $\tau^{-1}$. (**a**) The side view and (**b**) density distribution along *z* direction for the micelle nanostructure. (**c**) side view and (**d**) density distribution along the *y* direction for the vesicle nanostructure.

**Figure 6:**
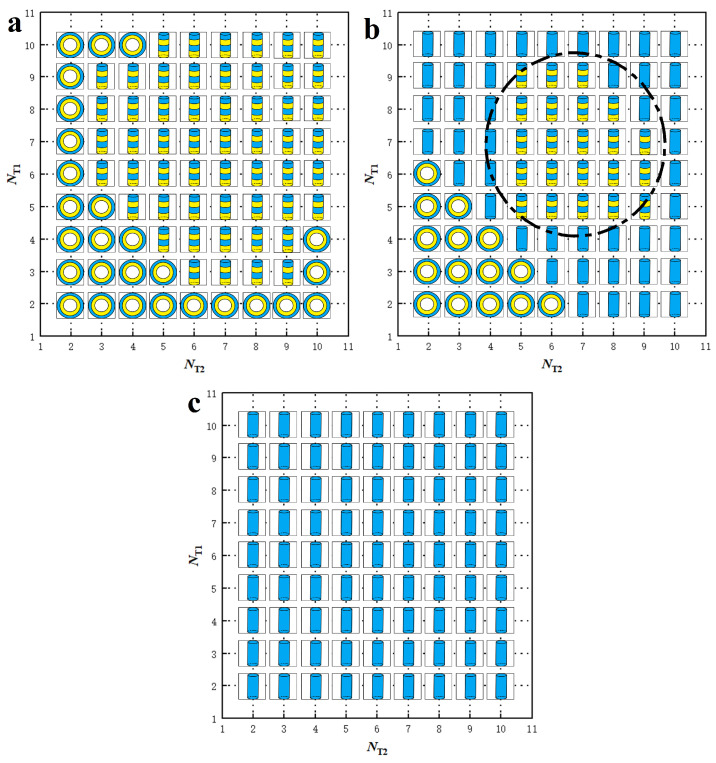
Phase diagrams for the nanostructures self-assembled from the lipid molecules in dilute solutions with ϕ=0.04 $r_{c}^{-3}$. (**a**) The zero shear flow with $\dot{\gamma }=0$ $\tau^{-1}$, (**b**) the weak shear flow with $\dot{\gamma }=0.073$ $\tau^{-1}$, and (**c**) the strong shear flow with $\dot{\gamma }=0.168$ $\tau^{-1}$. The phase diagrams are constructed based on the tail chain length of two types of lipid molecules, $N_{T1}$ and $N_{T2}$. The phase symbols 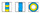 represent HD, micelle and vesicle nanostructures, respectively.

**Figure 7:**
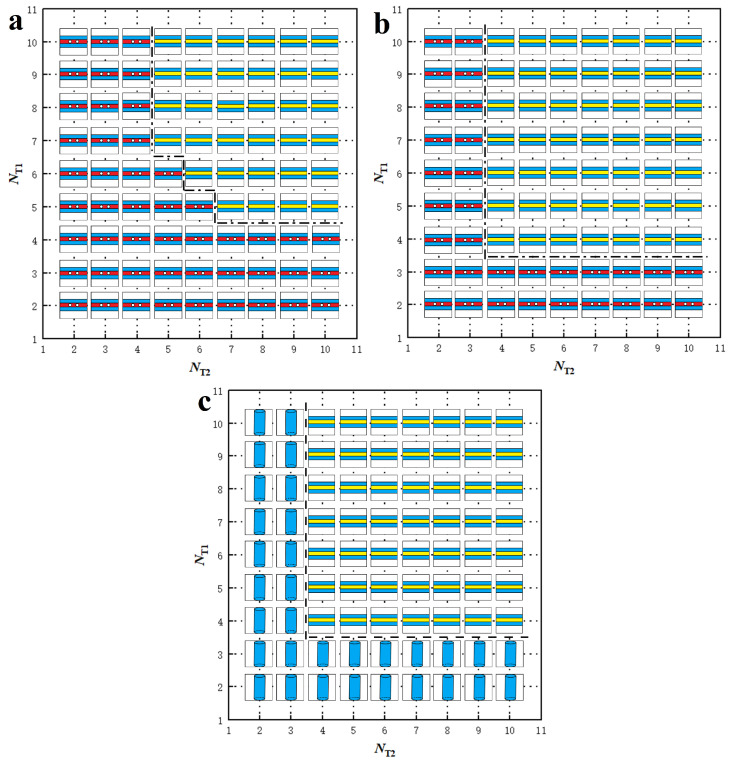
Phase diagrams for the nanostructures self-assembled from the lipid molecules in dense solutions with ϕ=0.08 $r_{c}^{-3}$. (**a**) The zero shear flow with $\dot{\gamma }=0$ $\tau^{-1}$, (**b**) the weak shear flow with $\dot{\gamma }=0.073$ $\tau^{-1}$, and (**c**) the strong shear flow with $\dot{\gamma }=0.168$ $\tau^{-1}$. The phase diagrams are constructed based on the tail chain length of two types of lipid molecules, $N_{T1}$ and $N_{T2}$. The phase symbols 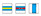 represent DL, PDL and micelle nanostructures, respectively.

**Figure 8:**
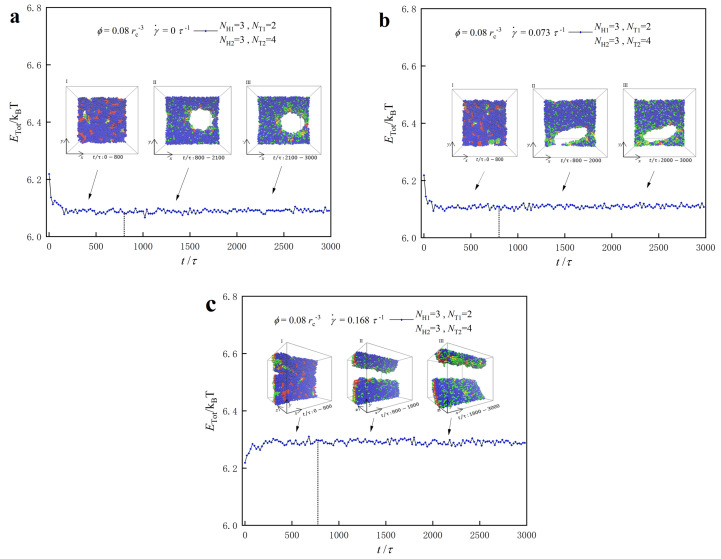
Representative dynamics processes for the lipid molecules in the dense solutions with ϕ=0.08 $r_{c}^{-3}$ under various shear flows. The system energies as functions of step time under (**a**) the zero shear flow with $\dot{\gamma }=0$ $\tau^{-1}$, (**b**) weak shear flow with $\dot{\gamma }=0.073$ $\tau^{-1}$, and (**c**) the strong shear flow with $\dot{\gamma }=0.168$ $\tau^{-1}$, respectively. The lipid molecule parameters are selected to be $N_{H1}=3,N_{H2}=3,N_{T1}=2$ and $N_{T2}=4$, and the representative structures are also inserted to illustrate the dynamics processes.

**Figure 9:**
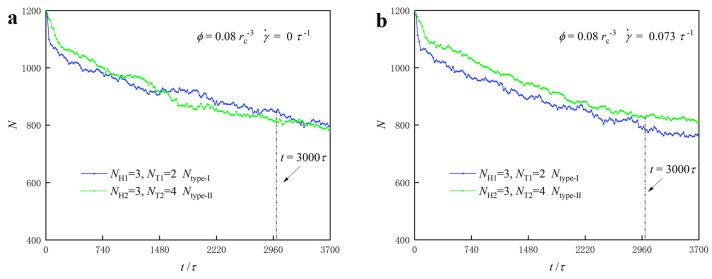
The mixture processes for the PDL structures with parameters of $N_{H1}=3,N_{H2}=3,N_{T1}=2$ and $N_{T2}=4$ under (**a**) the zero shear flow with $\dot{\gamma }=0$ $\tau^{-1}$ and (**b**) weak shear flow with $\dot{\gamma }=0.073$ $\tau^{-1}$, respectively. $N_{\mathrm{type}\text{-}I\;}$ denotes the particle numbers of type-I lipids in the right layer and $N_{\mathrm{type}\text{-}\mathrm{II}\;}$ is the particle numbers of type-II lipids in the left layer for the PDL bilayer, respectively.

**Figure 10:**
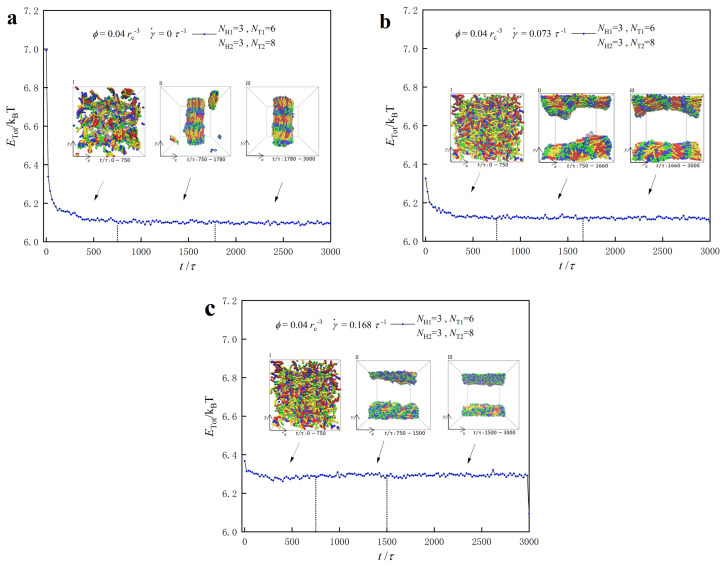
Representative dynamics processes for the lipid molecules in the dilute solutions with ϕ=0.04 $r_{c}^{-3}$ under various shear flows. The system energies as functions of step time under (**a**) the zero shear flow with $\dot{\gamma }=0$ $\tau^{-1}$, (**b**) weak shear flow with $\dot{\gamma }=0.073$ $\tau^{-1}$, and (**c**) the strong shear flow with $\dot{\gamma }=$ 0.168 $\tau^{-1}$, respectively. The lipid molecule parameters are selected to be $N_{H1}=3,N_{H2}=3,N_{T1}=6$ and $N_{T2}=8$, and the representative structures are also inserted to illustrate the dynamics processes.

**Figure 11:**
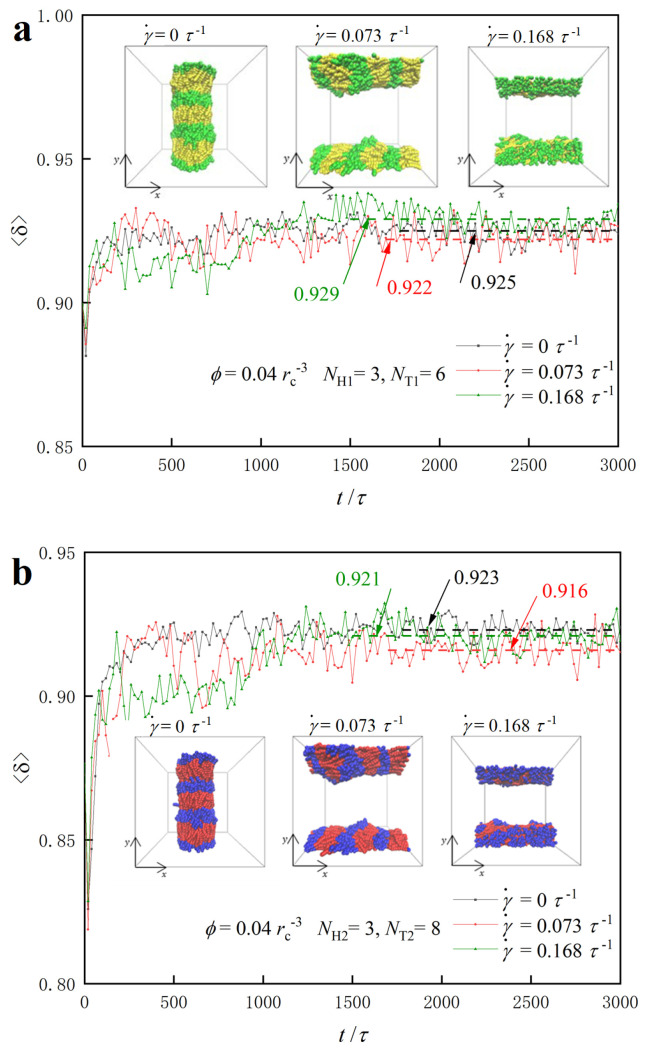
Representative dynamics processes for the lipid molecules in the dilute solutions with ϕ=0.04 $r_{c}^{-3}$ under various shear flows. The shape factors as functions of step time for the type-I lipid in (**a**) and type-II lipid in (**b**). The lipid molecule parameters are selected to be $N_{H1}=3$, $N_{H2}=3,N_{T1}=6$ and $N_{T2}=8$, and the representative structures are also inserted to illustrate the dynamics processes.

The parameter space for the lipid molecule system is huge. In our simulations, we investigated the self-assembly of lipid molecules in a solution by fixing certain parameters whilst varying others. We considered the self-assembly under zero shear flow, weak shear flow and strong shear flow cases by setting the shear rates to $\dot{\gamma }=0$ $\tau^{-1}$, 0.073 $\tau^{-1}$ and 0.168 $\tau^{-1}$, respectively. For each case, we examined both dilute and dense concentrations of lipid molecules with fixed lipid molecule numbers of n= 1200 and 2400, respectively. The head chain lengths were kept constant at $N_{H1}=N_{H2}=3$, whereas the lengths of the tail chains ( $N_{T1}$ and $N_{T2}$ ) were varied from 2 to 10 in all simulations. In Section 3.1, we analyze the nanostructures based on the distribution of particle density, as shown in Figure 3, Figure 4 and Figure 5. Subsequently, we organize these nanostructures into phase diagrams based on the chain lengths in Section 3.2, shown in Figure 6 and Figure 7. In Section 3.3, we discuss the corresponding dynamic processes using the system energy, particle number and shape factor, presented in Figure 8, Figure 9, Figure 10 and Figure 11.

### 3.1. Nanostructures

**Double layer (DL) and perforated double layer (PDL) structures**. The DL structure and PDL structure are depicted in Figure 3, illustrating the densities in 1D and 2D space, respectively. Figure 3a shows the density distributions of the DL structure in 2D space, with parameters $N_{H1}=N_{H2}=3,N_{T1}=6$ and $N_{T2}=4$ when ϕ = 0.08 $r_{c}^{-3}$ and $\dot{\gamma }=0$ $\tau^{-1}$. The head particles of both types of lipid molecules are adsorbed to the outer side of DL structures, whereas the tail particles are pushed into the inner side due to the amphiphilicity of lipid molecules in solutions. In general, the nanostructures are formed because of the competitive relationship between enthalpy and entropy of the system. For the lamellar structure, the interaction between phospholipid chains and water molecules leads to the separation of the hydrophilic and hydrophobic segments of the molecular chain due to their amphiphilicity and shear flows. However, the two blocks are linked to each other, and they cannot be completely separated due to the limitation of conformational entropy, leading to the formation of a layered structure. Additionally, a small amount of the head particles of the second type of lipid molecule is mixed with the head particles of the first type within the DL structure. To provide a clearer view, we plotted the 1D density profile in Figure 3b. Each $N_{H1},N_{T1},N_{H2}$ and $N_{T2}$ exhibits a single peak in its respective profile, located at z= 12.6 $r_{c}$, 13.8 $r_{c}$, 16.2 $r_{c}$ and 18.1 $r_{c}$, respectively, indicating the presence of a DL structure. The particle density profiles of double-layer structures were also reported in the previous work [61], where the structures were self-assembled from the mixture of lipids and phospholipids. Here, we used the density profiles to analyze the DL structures of lipids, which exhibit the double layers in the structures. We then observed the PDL structures, as shown in Figure 3c. Unlike DL, lipid molecules form a stable perforated membrane with small pores during self-assembly formation. Since lipid molecules are hydrophobic and hydrophilic in water solutions, they spontaneously assemble into a membrane with hydrophobic and hydrophilic layers. The formation mechanism of PDL is similar to that of DL. In order to maintain a similar hydrophilic contact area, the polymer chain tends to form pores to increase the hydrophilic contact area, thus achieving a rebalancing of entropy and enthalpy. In these structures, a single peak is observed in the tail particles $N_{T1}$ and $N_{T2}$, located at z=15.6 $r_{c}$ and 13.8 $r_{c}$, respectively. This is attributed to the hydrophobic nature of tail particles, which causes them to be pushed into the inner zones of the DL structure. However, double peaks appear in the $N_{H1}$ and $N_{H2}$ profiles. The main peaks in $N_{H1}$ and $N_{H2}$ are located at z=18.1 $r_{c}$ and 12.6 $r_{c}$, respectively, and the secondary peaks in $N_{H1}$ and $N_{H2}$ are located at z= 12.6 $r_{c}$ and 17.5 $r_{c}$, respectively. These results indicate that the main and secondary peaks overlap with each other, indicating a mixture of layer structures. The previous DPD simulations have also reported the DL structures of lipids in the absence of shear flows [44]. In the current simulations, we observed that the DL structures exist in the shear flows and the dense lipid concentrations. For the PDL mixing structure, however, further studies and explorations on the specific mechanism of pore formation are still needed. Previous research has suggested that well-penetrated membranes or DL structures play an important role in drug delivery in the field of medicine [80,81,82].

**Hierarchical disc (HD) structure**. We observed an interesting structure known as the HD structure, as shown in the side views of Figure 4. This particular HD structure is characterised by the parameters $N_{H1}=N_{H2}=3,N_{T1}=$ 6 and $N_{T2}=8$ when ϕ = 0.04 $r_{c}^{-3}$ and $\dot{\gamma }=0$ $\tau^{-1}$. In the HD structure, the disc structures overlap, forming a cylindrical shape, as shown in Figure 4. To investigate the distribution of particles, we plotted the particle density profiles along the *y*-direction for the lipid molecules in Figure 4b. The density profiles of $N_{H1},N_{T1},N_{H2}$ and $N_{T2}$ are denoted with various colours. The density profiles exhibit three similar regions, namely, 0 c≤ y < 10 $r_{c}$, 10 $r_{c}\leq$ y < 20 $r_{c}$ and 20 $r_{c}\leq$ y < 30 $r_{c}$, indicating that the disc has a thickness of 10 $r_{c}$. In particular, within the region of 0 $r_{c}\leq$ y < 10 $r_{c}$, both $N_{H1}$ and $N_{H2}$ exhibit peaks at the interfaces between two discs, whereas the peaks of $N_{T1}$ and $N_{T2}$ appear in the middle zones. This observation suggests that the disc structure resembles the DL structure, possessing double layers but confined within a cylinder. To provide a more detailed description of this HD structure, we plotted the top view of the structure in the *x*–*z* plane in Figure 4c. The top view clearly shows that the particle distributions are confined within a circular zone. Furthermore, we plotted the particle density along the *z*-direction in Figure 4d. The particles are mainly distributed between z=7 $r_{c}$ and z=21 $r_{c}$ and exhibit the same trend of variation with peaks in the middle zone. We note that the hierarchical structures, such as the hierarchical cylinders, have been reported in the previous works [83,84,85,86,87,88]. Generally, the hierarchical cylinders are easier to form under the cylindrical confinements [83,84,85]. The HD observed in the lipid mixture is somewhat different, which indicates that the solution conditions can also provide similar effects to confinements. One common condition is when the polymer is confined within cylindrical pores, where adsorption interactions or entropic effects arising from the pore confinement induce the formation of disc structures [83,84,85]. Additionally, hierarchical structures can be observed in bulk systems when polymers possess special topologies, such as star polymers [86]. Moreover, similar striated columnar micelles have been observed in water, where surfactant heads and tails are alternately arranged along the column axis [88]. In our study, we observe the formation of HD structures in lipid molecules due to their amphiphilicity in solutions. Specifically, HD exhibits a cylindrical shape, resulting from an asymmetry in the length of hydrophilic head and hydrophobic tail chains. However, this kind of cylinder is composed of layers, which is different from the common cylinder structure. In fact, the formation of an HD structure is not only related to the chain asymmetry, but also to the shear flows and the concentration of the polymer chain, which will be discussed in the phase diagram.

**Micelles and vesicles**. In the solutions, we observed the micelles and vesicles with lipid molecules, as shown in Figure 5, where the parameters are $N_{H1}=N_{H2}=3$, $N_{T1}=2$, $N_{T2}=8$ for the micelles when ϕ = 0.08 $r_{c}^{-3}$, $\dot{\gamma }=0$ $\tau^{-1}$ and $N_{H1}=$$N_{H2}=3,N_{T1}=2$, $N_{T2}=10$ for the vesicles when ϕ = 0.08 $r_{c}^{-3}$, $\dot{\gamma }=0.073$ $\tau^{-1}$. For the micelle structure, the head particles of the two types of lipid molecules are shown in blue and green, and the tail particles are shown in red and yellow. The tail particles are encapsulated within the head particles because lipid molecules in aqueous solution are amphiphilic. To provide a clear visualization of the structure, we plotted the density profiles along the *z*-direction in Figure 5b. The profiles of $N_{H1},N_{T1}$ and $N_{H2}$ exhibit double peaks, whereas the $N_{T2}$ profile displays a single peak. This indicates that the short chains form double layers at the interfaces, whereas the longer hydrophobic chains are pushed into the central zone of micelles. Although the micelle structure was also reported in the previous work [61], the micelle structure observed in the current simulations is under shear flow conditions. A similar case is observed for the vesicle structures, as shown in Figure 5c,d. Specifically, the $N_{T2}$ profile reaches its highest value at y=16 $r_{c}$, corresponding to the central part of the vesicle structure, as shown in Figure 5d. In addition, the density profile shows that this vesicle structure is asymmetric. The previous works have also reported the asymmetric vesicles in the other polymer systems due to several factors such as the different types of polymers [8,56,61]. The current DPD observations suggested that the asymmetry about the vesicles may have originated from the different tail chain lengths for the two types of lipids. In particular, we note that the shear flow and polymer concentration also play a very important role in the formation mechanism for the micelle and vesicle, as well as for the HD, PDL and DL structures, in addition to their head and tail chain asymmetry.

### 3.2. Phase Diagrams

Phase diagrams provide valuable information on phase transitions involved in lipid molecule self-assembly, a phenomenon extensively studied in phospholipid systems [14,50,56]. In our simulations, we organize the observed phases into phase diagrams based on the tail lengths of lipid molecules, $N_{T1}$ and $N_{T2}$. We construct six phase diagrams corresponding to different lipid concentrations, *n*, and shear rates, $\dot{\gamma }$. We determine the concentration ϕ by calculating the ratio between the lipid chain number and simulation box volume, i.e., ϕ=n/V. When the number of chains *n* is 1200, the concentration ϕ is 0.04 $r_{c}^{-3}$; when *n* is 2400, the concentration ϕ is 0.08 $r_{c}^{-3}$. The definition of polymer concentration is also relative; in general, we can observe continuous structures such as layers in dense solutions. We obtained stable phase diagrams in dilute solutions (ϕ = 0.04 $r_{c}^{-3}$) and dense solutions (ϕ = 0.08 $r_{c}^{-3}$) under zero ($\dot{\gamma }=0$ $\tau^{-1}$), weak ($\dot{\gamma }=0.073$ $\tau^{-1}$), and strong shear ($\dot{\gamma }=0.168$ $\tau^{-1}$) conditions, respectively, as shown in Figure 6 and Figure 7.

For the dilute lipid molecule concentration ϕ=0.04 $r_{c}^{-3}$, we observe three phases: HD, micelles and vesicles. We construct phase diagrams for these phases in the zero, weak and strong shear flows, as shown in Figure 6a–c. The phase diagrams are based on the tail chain lengths, $N_{T1}$ and $N_{T2}$, ranging from 2 to 10 with a step of 1. Two general characteristics can be highlighted for these phase diagrams. Firstly, the diagrams exhibit symmetry about the diagonal line due to the equivalence between $N_{T1}$ and $N_{T2}$. Usually, the theoretical or simulated phase diagram is different from the experimental phase diagram. A typical example is the phase diagram of the diblock copolymer, in which the theoretical simulation of the phase diagram based on the block ratio and Flory–Huggins coefficient is symmetrical, while the experimental phase diagram is somewhat asymmetrical. This may be because the experimental conditions are more complicated. Similar symmetric phase diagrams have been reported in other polymer systems, both in bulk and under confinement, for similar reasons [44,56,89,90]. In our case, we observe symmetric phase diagrams in lipid solutions. Secondly, vesicles are only present in the zero or weak shear flows, whereas cylindrical structures, such as HD or micelles, appear over a wide range of shear flows. Specifically, the HD structure is observed exclusively in zero shear flow and transitions partially into weak shear flows (Figure 6b) and completely into strong shear flows (Figure 6c). This indicates that vesicle and HD structures are unstable under shear flows and can deform into cylindrical structures. The alignment of the cylinders along the direction of shear flow is a logical result because it is also observed in phase transitions induced by electric fields, where cylinders align along the direction of the electric fields [91,92].

We then consider the special characteristics of the phase diagrams in the zero, weak, and strong shear flows. In the zero shear flow, we observe two structures, HD and vesicles, as shown in Figure 6a. The HD structures are distributed in the phase space where the tail chains are long, whereas the vesicles predominantly appear in the phase space with shorter tail chain lengths. However, we also observe that vesicle structures can form with strong asymmetric tail chains when $N_{T1}$ is larger than $N_{T2}$ or vice versa, as shown in Figure 6a. This asymmetry disappears in the weak shear flows, as shown in Figure 6b. In the weak shear flows, vesicles with long tail chains and HD structures partially transition into micelles with cylindrical symmetry, induced by the shear flows. With increased shear rate, we only observe one structure, the micelle with cylindrical symmetry, in the strong shear flows, as shown in Figure 6c. This indicates that even vesicles with short tail chain lengths are unstable under strong shear flows, which is consistent with previous observations [14,61].

For the dense lipid molecule concentration (ϕ=0.08 $r_{c}^{-3}$), we observe three phases: DL, PDL and micelles, in the corresponding phase diagrams for zero, weak and strong shear flows, as shown in Figure 7a–c. Similar to the dilute concentration cases, the phase diagrams exhibit symmetry about the diagonal lines due to the equivalence between $N_{T2}$ and $N_{T2}$ in all three shear flows. However, noticeable differences are observed between the dilute and dense concentration cases. Firstly, lamellar structures, DL and PDL, appear in zero flows in dense lipid concentrations, as shown in Figure 7a. DL occupies the region with larger $N_{T2}$ and $N_{T2}$, whereas PDL occupies the region with relatively smaller $N_{T2}$ and $N_{T2}$. Our observations are similar to previous simulations involving lipid molecules with one head chain and two tail chains [50]. This can be explained by the fact a larger number of lipid molecules facilitates the assembly into DL structures, and the pores in PDL result from insufficient lipid molecules. Secondly, we observe that the pores can be repaired by the shear flows, as shown in Figure 7b, where the phase regions of DL structures are slightly enlarged. This suggests that the PDL structure is slightly unstable under shear flows. The pores in PDL are influenced by multiple factors, such as lipid concentration and topology [50,61]. In the strong shear flows, PDL completely disappears and transitions into the micelle structure due to the shear forces, as shown in Figure 7c. In the strong shear flows, we observe the stability of DL structures, indicating that shear flow facilitates the formation of membranes in experiments.

Here, we summarize the effects of polymer concentration and shear flow on the phase diagrams. First, the polymer chain tends to self-assemble into the HD, micelle and vesicle structures in the dilute concentration solutions. In dense concentrations, the phospholipid molecules tend to self-assemble into layered and perforated layered structures since they require more polymer chains to participate in self-assembly. Secondly, it is difficult for the vesicles and HD structures to exist under strong shear flow, because the shear force tends to elongate these structures along the direction of the shear force, subsequently them transforming into cylindrical or layered structures.

### 3.3. Dynamic Processes

In the previous subsections, we examined the equilibrium structures and phase diagrams of lipid molecule self-assembly. In this subsection, we investigate the dynamic processes to understand the formation mechanisms of these structures. We focus on two specific cases: a dense lipid concentration (ϕ=0.08 $r_{c}^{-3}$) with parameters $N_{T1}=2$ and $N_{T2}=4$, and a dilute lipid concentration (ϕ=0.04 $r_{c}^{-3}$) with parameters $N_{T1}=6$ and $N_{T2}=8$. Figure 8 and Figure 9 depict the dynamic processes for the dense concentration case, and Figure 10 and Figure 11 show the dynamic processes for the dilute concentration case.

For the phase point with $N_{T1}=2$ and $N_{T2}=4$ in the dense lipid concentration case, we observe the self-assembly of lipid molecules into PDL structures under zero and weak shear flows, whereas micelles with cylindrical symmetry form under strong shear flows. Figure 8 illustrates the system energies as functions of step time for the three shear flows, along with representative structures. According to the variations in energy and the self-assembled structures, as well as the variations of particle numbers that will be described in Figure 9, we divided the dynamics processes into two stages in a simple way. First, we concentrated on the dynamics process of pore formation in the lipid membranes under the zero shear flows, as shown in Figure 8a. In the initial stage of *t* = 0–800 τ, the system energy decreases rapidly and the pore begins to form in this stage. Commonly, the initial irregular distributions of molecules lead to obvious variations in system energies, such as the observations in the phospholipid molecules [56]. Then, the energy decreases much more slowly in the adjustment stage and subsequently reaches a relatively stable value. These two stages differ from the dynamics processes observed in the other lipid systems [14,44,50,61], where the various types of lipid molecules were modeled and three stages were observed. We note that the pore continues to its position and size, although the energy changes very slowly in the adjustment stage (t=800–3000 τ), as shown in the insets of Figure 8a. This indicates ongoing adjustments to both types of lipid molecules in the aqueous solution to form the PDL structure. Finally, lipid molecules assemble into stable PDL structures, in which the amphiphilic interaction between the lipid molecule’s heads and tails drives self-assembly into PDL structures. The dynamic processes of PDL under zero shear flow are similar to those observed in biological systems with PDL [93].

Similar evolution processes are observed under weak and strong shear flows, as shown in Figure 8b,c. The initial stages occur within t=0–800 τ, followed by adjustment stages within t=800–3000 τ under weak and strong shear flows. However, the shear flow influences the morphology of pores appearing in the PDL adjustment stage, illustrating the effect of flow shear on the self-assembly process of lipid molecules [14,61]. For the strong shear flow (Figure 8c), the self-assembly morphology of lipid molecules transitions from PDL to micelle structures, indicating a strong influence of shear flow on the self-assembly process. Previous simulation results also indicate that the self-assembly formation stages for other types of lipid molecules become progressively shorter under strong shear flows, accelerating the self-assembly process [61]. PDL structures exhibit weaker stability compared with micelle structures, as mentioned in the phase diagram in Section 3.2.

We shift our focus to the dynamic processes of PDL structures under zero and weak shear flows in the dense lipid concentration case, as shown in Figure 9. We observe alternating mixing between the two types of lipid molecules. To describe the mixing phenomenon, we plotted the number of chains for each type of lipid molecule as functions of step times under zero shear rate $\left(\dot{\gamma }=0\right)$ and weak shear rate $\left(\dot{\gamma }=0.073\right)$ $\tau^{-1}$ in Figure 9a and Figure 9b, respectively. The first type of lipid (type-I) is represented by the green curve, and the second type (type-II) is represented by the blue curve. We then counted the numbers of type-I lipid chains in the right layer and type-II lipid chains in the left layer, and we present the results in Figure 9a and Figure 9b, respectively.

At $\dot{\gamma }=0$ $\tau^{-1}$ and t=0 τ, the right layer of the PDL structure contains 1200 type-I and 0 type-II lipid molecule chains, whereas the left layer of the PDL structure contains 0 type-I and 1200 type-II lipid molecule chains, as shown in Figure 9a. The results demonstrate that the type-I lipid molecules in the right layer continuously transfer to the left layer. Conversely, the type-II lipid molecules in the left layer transfer into the right layer during the dynamic processes. More detailed data reveal that the number of type-I lipid molecules decreases from 1200 to 1017 in the right layer, and the number of type-II lipid molecules in the left layer decreases from 1200 to 1024 at t= 800 τ. During the adjustment stage, we observe a continuous decrease in the number of type-I lipid molecules in the right layer and type-II lipid molecules in the left layer. At t= 3000 τ, the number of type-I lipid molecules in the right layer remains at 865, and the number of type-II lipid molecules in the left layer decreases to 877. The detailed data show that the particle numbers of type-I and type-II lipids decrease more obviously in the initial stages than those in the adjustment stages, indicating the more obvious mixing in the initial stages. It is reasonable to assume that there is a stable mixing near t= 3000 τ, as shown in Figure 9. Our findings regarding the mixture in PDL are consistent with previous studies where shear flow is absent [44]. We also observe similar mixing processes under weak shear flows, as shown in Figure 9b. The shear flow can affect the degree of mixing in the dynamic processes. Specifically, the number of type-I lipid chains decreases by 371, and the number of type-II lipid chains decreases by 352 throughout the whole stage. This reinforces the effect of shear on the dynamic process of lipid molecule self-assembly, making the mixing of the two lipid molecules more apparent.

We then examine the dynamic processes of lipid molecules in a dilute concentration of ϕ=0.04 $r_{c}^{-3}$, taking the phase point with parameters of $N_{T1}=6$ and $N_{T2}=8$ as an example. We plot the system energies as functions of step time in Figure 10, considering zero, weak and strong shear flows. Unlike the case in the dense lipid concentrations, the self-assembly of lipid molecules in dilute concentrations also undergoes three stages in the dynamic processes. In the case of zero shear flow, as shown in Figure 10a, we observe the initial stage from 0 τ to 750 τ, the adjustment stage from 750 τ to 1780 τ, and the stable stage from 1780 τ to 3000 τ, during which the molecules self-assemble into the HD stable structure oriented along the *y*-axis. In the weak shear case, shown in Figure 10b, the dynamics process still consists of three stages, and the stable structure remains HD. However, the weak shear flow changes the orientation of HD to the *x*-direction. In this case, the shear flow also shortens the duration of the adjustment stage, which spans from 750 τ to 1660 τ. Under the strong shear flow, shown in Figure 10c, the lipid molecules self-assemble into another micellar structure, which also aligns with the direction of the shear flow.

To investigate the conformational transitions of lipid molecules, we consider the shape factor in the dynamics processes. The shape factor can be expressed as [94,95]

(10) $$\langle \delta \rangle =1-3\langle \frac{L_{1}^{2}L_{2}^{2}+L_{2}^{2}L_{3}^{2}+L_{1}^{2}L_{3}^{2}}{{\left(L_{1}^{2}+L_{2}^{2}+L_{3}^{2}\right)}^{2}}\rangle ,$$

where $L_{1}^{2},L_{2}^{2}$ and $L_{3}^{2}$ are three eigenvalues of the gyration radius tensor $R_{g}^{2}$, which is defined by Equation (11),

(11) $$R_{g}^{2}=\left(\begin{array}{ccc} R_{gxx}^{2} & R_{gxy}^{2} & R_{gxz}^{2}\\ R_{gyx}^{2} & R_{gyy}^{2} & R_{gyz}^{2}\\ R_{gzx}^{2} & R_{gzy}^{2} & R_{gzz}^{2} \end{array}\right),$$

where the elements $R_{g\alpha \beta }^{2}$ are given by Equation (12),

(12) $$\langle R_{g\alpha \beta }^{2}\rangle =\frac{1}{N}\sum_{i=1}^{N}\langle \left(r_{i,\alpha }-r_{c,\alpha }\right)\left(r_{i,\beta }-r_{c,\beta }\right)\rangle ,\alpha ,\beta \in \left\{x,y,z\right\},$$

where *N* represents the number of particles, and $r_{i,x}$ in brackets denotes the *x* coordinate of the *i*-th particle. In particular, the conformation tends to be spherical when 〈δ〉=0. The chain exhibits a circular conformation when 〈δ〉=0.5, and the chain exhibits a rod-like conformation when 〈δ〉=1.0.

We plot the shape factors as functions of step time for the two types of lipid molecules, where the polymer parameters are $N_{H1}=N_{H2}=3,N_{T1}=6$, and $N_{T2}=8$ under various shear flows, as shown in Figure 11. The results show that the average shape factor of both types of lipid molecules stabilizes at approximately 0.92, indicating that their shapes at the final stable stages under different shear conditions resemble rod-like structures. Specifically, the shape factor of the lipid molecule initially increases slightly and then plateaus, ultimately reaching approximately 0.92 under zero, weak and strong shear flows. In particular, the average value of the shape factor for the first class of lipids is 0.925 at $\dot{\gamma }=0$, 0.922 at $\dot{\gamma }=0.073$ $\tau^{-1}$ and 0.929 at $\dot{\gamma }=0.168$ $\tau^{-1}$, as shown in Figure 11a. The inserted structures in Figure 11a directly show the molecular arrangement, where the lipid molecules self-assemble into structures aligned in the *y*-axis direction under zero shear flow. However, when subjected to strong shear and weak shear flows, the lipid molecules self-assemble into structures along the *x*-axis direction. As the shear rate increases, the shape of the lipid molecules gradually approaches a rod-like shape, increasing the corresponding shape factor. This is consistent with the observation that shape factor values are higher under strong shear conditions compared with weak shear conditions [50]. We also plot the shape factors for the type-II lipid molecules $\left(N_{H2}=3,N_{T2}=8\right)$ in the dynamics processes, as shown in Figure 11b. The results show that the shape factor of type-II lipid molecules follows a similar trend to that of type-I lipid molecules. However, the data indicate that the type-II molecules have relatively smaller shape factor values compared with those of type-I molecules. This discrepancy arises from the relatively shorter tail chains in type-I lipid molecules, which more easily adopt rod-like conformations compared with type-II molecules. Similarly, the shear rate can affect the mean value of the shape factors for type-II lipid molecules, with a shape factor of 0.916 at $\dot{\gamma }=0.073$ $\tau^{-1}$ and 0.921 at $\dot{\gamma }=0.168$ $\tau^{-1}$.

## 4. Summary

In this study, we performed DPD simulations to investigate the effects of shear flows on the self-assembly of lipid molecules in solutions. We investigated both dilute and dense lipid concentrations under zero, weak and strong shear flows. Various nanostructures emerged under these shear conditions, and we categorized them into phase diagrams based on the lipid tail lengths. We examined the dynamics processes of specific nanostructures through system energies, particle numbers and shape factors.

We observed the formation of DL, PDL, HD, micelle and vesicle structures under zero, weak and strong shear flows. The identification of these structures was based on particle densities, with the HD structure being a novel hierarchical disc structure observed for the first time in lipid systems. Moreover, we observed the PDL structure, which is a mixed structure where two types of lipid molecules are present in two distinct layers. We organized these nanostructures into six phase diagrams for dilute and dense lipid solutions. The phase diagrams showed symmetry along the diagonal line due to the equivalence of $N_{T}1$ and $N_{H}2$. In dilute lipid concentrations, vesicles were only present under zero or weak shear flows, whereas cylindrical structures, such as HDs or micelles, appeared across a wide range of shear flows. HD structures were exclusively observed under zero shear flows. For dense lipid concentrations, we observed lamellar structures (DL and PDL) under zero shear flows. DL occupied the region with large $N_{T}1$ and $N_{T}2$, whereas PDL occupied the region with relatively small $N_{T}1$ and $N_{T}2$. We also observed that shear flows can repair pores, indicating slight instability of the PDL structure under shear. Under strong shear flows, the PDL completely transformed into the micelle structure due to shear forces. By contrast, the DL structure remained stable, suggesting the potential use of shear flows for membrane preparation in experimental settings. In both cases, shear flows induced phase transitions and realigned structure directions.

We then examined the dynamics of structural formation through several examples. In the dilute and dense lipid concentrations, we observed two or three stages in the dynamics processes, which are different due to the lipid concentrations. The system energies revealed that the evolution processes were similar under zero, weak and strong shear flows, but the stage periods varied because of the effect of shear flows, which accelerated the self-assembly process. In dense lipid concentrations, we focused on the dynamic processes of PDL structures under zero and weak shear flows. We observed alternating mixtures of the two types of lipid molecules. Type-I lipid molecules continuously transferred from the right layer to the left layer, and shear flow affected the degree of mixing, making the two lipid molecules more prominently mixed. At dilute lipid concentrations, we examined the conformational transitions of lipid molecules using shape factors, focusing on the HD structure. The results showed that the average shape factor of both types of lipid molecules stabilized at approximately 0.92, indicating rod-like structures at the final stable stages under different shear conditions. As the shear rate increased, the lipid molecules adopted a more rod-like shape, increasing the corresponding shape factor. These findings contribute to our understanding of the biomolecular self-assembly in a solution and have potential applications in biomedicine.

## Figures and Tables

**Table 1:** The system parameters involved in the simulations.

The unit mass	*m*	The unit energy	$k_{B}T$
The unit time	τ=1.88 ns	Particle number density	$\rho =\frac{N}{L_{x}\times L_{y}\times L_{z}}$ = 3 $r_{c}^{-3}$
A DPD particle volume	$V_{P}=0.03$ $\;{\mathrm{nm}}^{3}$	Weak shear flows	$\dot{\gamma }=0.073$ $\tau^{-1}$
The unit length	$r_{c}=0.5$ nm	Strong shear flows	$\dot{\gamma }=0.168$ $\tau^{-1}$

**Table 2:** The interaction parameters in the simulations.

Box size $V=L_{x}\times L_{y}\times L_{z}$ = 30 $r_{c}$ · 30 $r_{c}$ · 30 $r_{c}$							
DPD parameters σ = 3.0 γ = 4.5							
	$a_{ij}$	Beads	$H_{1}$	$T_{1}$	W	$H_{2}$	$T_{2}$
	Beads						
$H_{1}$			25				
$T_{1}$			100		25		
W			40	100	25		
$H_{2}$			25	100	40	25	
$T_{2}$			100	100	100	100	25

## Data Availability

Not applicable.
